# A new species of the genus *Disogmus* Förster (Hymenoptera, Proctotrupoidea, Proctotrupidae) from the Eocene Rovno amber

**DOI:** 10.3897/zookeys.130.1560

**Published:** 2011-09-24

**Authors:** V. Kolyada, E. Perkovsky

**Affiliations:** 1 Borissiak Paleontological Institute, Russian Academy of Sciences, Profsoyuznaya St. 123, Moscow, 117997 Russia; 2 Schmalhausen Institute of Zoology, Bogdan Khmelnitski str. 15, Kiev, 01601 Ukraine

**Keywords:** Proctotrupidae, Disogmus, Late Eocene, Rovno amber, Ukraine

## Abstract

*Disogmus rasnitsyni* Kolyada & Perkovsky, sp. n. is described from a fossil inclusion of Late Eocene amber (Ukraine). The new species is most similar to *Disogmus basalis* (Thomson, 1857), in particular, in the shape of the tyloids and the general shortening of the segments of antennae, but distinctly differs from it and the other species of the genus by having tyloids on flagellar segments 2–4 compared to 3–6 and 4–7 in other species.

## Introduction

The Proctotrupidae is a relatively small, cosmopolitan family of parasitic wasps which prefer regions with temperate and humid climate. They are most diverse in the Holarctic, where they occur predominantly in the forest zone. Proctotrupidae parasitize the larvae of beetles and dipterans or, more rarely, larvae of the lepidopteran family Oecophoridae. In the modern fauna, the family comprises about 320 species classified into 27 genera (Townes 1981; Johnson 1992).

In the Cenozoic, Proctotrupidae are represented by the extant genera *Fustiserphus* Townes, 1981, *Mischoserphus* Townes, 1981, and *Oxyserphus* Masner, 1961 and are known from the Late Eocene Baltic and Rovno amber (Kolyada and Mostovski 2007). Moreover, from the Florissant locality (USA), currently dated as the uppermost Eocene (Evanoff et al. 2001), in addition to *Mischoserphus* and *Oxyserphus*, the genus *Nothoserphus* Brues, 1940 is known (Kolyada 2009). Unidentified proctotrupids are known from the Middle Eocene deposits in Washington State and in the Middle Eocene Kishenehn Formation in northwestern Montana (USA) as well as in British Columbia, Canada (Okanagan Highlands).

From the Rovno Amber previously there was known only one species of Proctotrupidae: *Oxyserphus obsolescens* (Brues, 1940) (Kolyada and Mostovski 2007). Rovno amber and its arthropod fauna is described in more details by Perkovsky et al. (2007).The present article describes a new species of genus *Disogmus*, which was previously unknown in the palaeontological record.

## Material and methods

Material used in this study is deposited in the amber collection of the Schmalhausen Institute of Zoology of National Academy of Sciences of Ukraine, Kiev (SIZK). Pictures were taken through a Leica M 165 microscope with Leica DFC425 digital camera. To create diffused illumination, a cup of white styrofoam was placed between an object and a light source. The captured images were assembled with Helicon Focus 5.01 software and edited in Helicon Filter 4.7 and Adobe Photoshop CS4.

## Systematics

### Family PROCTOTRUPIDAE Latreille, 1802

**Subfamily PROCTOTRUPINAE Latreille, 1802**

**Tribe DISOGMINI Kozlov, 1970**

#### Genus Disogmus

Förster

http://species-id.net/wiki/Disogmus

Disogmus Förster, 1856: 99. Type species: *Proctotrupes areolator* Haliday, designated by Walker (1874), Ashmead (1893). Key to species.

##### Diagnosis.

Front wing 1.8–3.5 mm, long. Body moderately slender. Head clearly transverse, rounded, frons convex. Apical margin of clypeus simple and convex. Mandible moderately stout, with a single point. Cheeks usually with sulcus from eye to mandible. Occipital carina developed, but not reaching hypostomal sulcus. Male flagellum with noticeable tyloids. Pronotum with strong angular pronotal shoulder that is surmounted by a sharp carina. Epomia present. Scutellar pit without inner longitudinal carinae. Notaulus varying from about 0.5 as long as tegula to quite long and reaching beyond center of mesoscutum. Horizontal mesopleural groove complete and strong. Stigma small, r-rs (vertical part of radius) about 3.0 times as long as wide, radial vein runs from apical 0.3 of stigma. Radial cell long, the side next to costa about 2.8 times as long as depth of stigma. Hind spur is equal to 0.3 of the length of basitarsus or even shorter. Abdomen with stalk about 1.2 times as long as high. Ovipositor sheath 0.7-0.9 times as long as hind tibia, smooth, slender, evenly curved, gradually tapered to a rounded apex, with some erect hairs that are denser near apex.

##### Distribution.

This small genus comprises 5 described species, 3 species of which inhabit the Holarctic and the other 2 species occur only in the Nearctic (Townes 1981, Buhl 1998). Moreover, we have found this genus in Mexico and Taiwan. The specimens of this genus presumably parasitize the larvae of Sciaridae (Sciaroidea, Diptera) (Townes 1981). Sciarids are overrepresented in Rovno amber in comparison with Baltic amber (Perkovsky et al. 2007).

#### Disogmus rasnitsyni

Kolyada & Perkovsky sp. n.

urn:lsid:zoobank.org:act:4313A2CD-F188-48D0-88A7-7FC3CF0C6CF2

http://species-id.net/wiki/Disogmus_rasnitsyni

Figs 1–2

##### Holotype.

♂, SIZK K-3806, Klesov, Rovno amber, Late Eocene. Syninclusions: Diptera (Chironomidae and Tipuloidea), Tipuloidea.

##### Etymology.

Named in honor of the paleoentomologist Prof. Alexander Rasnitsyn.

##### Description.

Length of body 2.5 mm; length of forewing 1.5 mm. Antenna short, length to width ratio of 1, 2 and 9 flagellomeres as 2.5:1.0; 1.7:1.0; 1.3:1.0. Tyloids on flagellar segments 2–4, the tyloids in form of subtriangular slaws that is about 0.3 as long at base as the segments are long (Fig. 2). Side of collar weakly rugulose. Epomia not interrupted. Notaulus quite long, reaching beyond center of mesoscutum. Stigma narrow, r-rs (vertical part of radius) about 3.4 times as long as wide. Radial cell long, side next to costa about 0.8 as long as radius. Mesopleuron below tegula with some fine horizontal wrinkles. Metapleuron rugose, with a small but distinct carina from its upper front part to anterolateral edge of propodeum.

**Figures 1–2.**
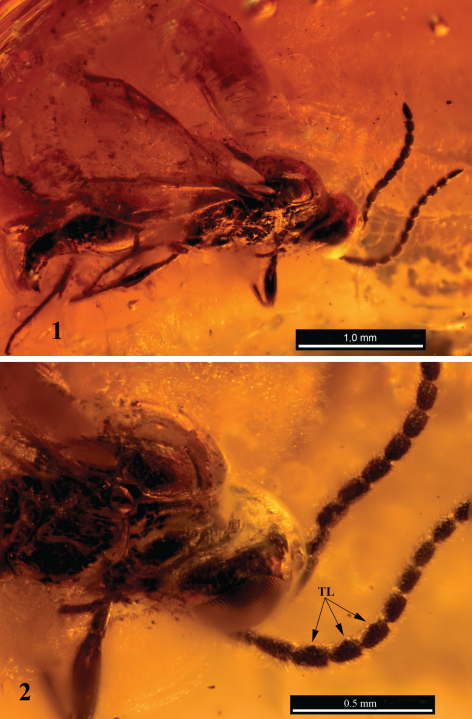
*Disogmus rasnitsyni*, sp. n., male holotype. **1** body, dorsolateral view **2** tyloids (TL) on flagellar segments.

##### Comparison.

Within the genus *Disogmus*, the new species is most similar to *Disogmus basalis* (Thomson, 1857), in particular, by the shape of tyloids and the general shortening of the segments of antennae. The new species distinctly differs from *Disogmus basalis* and the other species of the genus by having tyloids on flagellar segments 2-4 compared to 3–6 and 4-7 that other species have.

## Supplementary Material

XML Treatment for Disogmus

XML Treatment for Disogmus rasnitsyni
